# Dielectrophoresis Testing of Nonlinear Viscoelastic Behaviors of Human Red Blood Cells

**DOI:** 10.3390/mi9010021

**Published:** 2018-01-09

**Authors:** Yuhao Qiang, Jia Liu, E Du

**Affiliations:** Department of Ocean and Mechanical Engineering, Florida Atlantic University, Boca Raton, FL 33431, USA; yqiang2015@fau.edu (Y.Q.); jliu2015@fau.edu (J.L.)

**Keywords:** biomechanics, viscoelasticity, red blood cells, dielectrophoresis, microfluidics

## Abstract

Dielectrophoresis in microfluidics provides a useful tool to test biomechanics of living cells, regardless of surface charges on cell membranes. We have designed an experimental method to characterize the nonlinear viscoelastic behaviors of single cells using dielectrophoresis in a microfluidic channel. This method uses radio frequency, low voltage excitations through interdigitated microelectrodes, allowing probing multiple cells simultaneously with controllable load levels. Dielectrophoretic force was calibrated using a triaxial ellipsoid model. Using a Kelvin–Voigt model, the nonlinear shear moduli of cell membranes were determined from the steady-state deformations of red blood cells in response to a series of electric field strengths. The nonlinear elastic moduli of cell membranes ranged from 6.05 µN/m to up to 20.85 µN/m, which were identified as a function of extension ratio, rather than the lumped-parameter models as reported in the literature. Value of the characteristic time of the extensional recovery of cell membranes initially deformed to varied extent was found to be about 0.14 s. Shear viscosity of cell membrane was estimated to be 0.8–2.9 (µN/m)·s. This method is particularly valuable for rapid, non-invasive probing of mechanical properties of living cells.

## 1. Introduction

Mature red blood cells (RBCs) in humans consist of cell membrane and cytoplasm, mainly hemoglobin. Its unique bi-concave disc shape, membrane viscoelastic properties, and cytoplasmic viscosity are the primary factors that affect the ability of a RBC to flow through the microvasculature. Characterization of the mechanical properties of RBC membranes has allowed us to better understand and accurately model the physiological processes, such as blood rheology [1], splenic filtration [2], cell adhesion [3], and cell-cell interactions [4]. Viscoelasticity of RBC membranes is of particular interest for the studies of blood diseases [5]. 

Microfluidics has been recognized as a versatile tool to develop experimental models [6] for characterization of cell biomechanics and rheology, complementary to those classical physics and engineering techniques [7]. Passive shear flow [3] and dielectrophoresis (DEP) [8] are the two main mechanisms implemented in microfluidic environment for the biomechanical studies of living cells. The DEP polarization force [9] exerted on a cell is along with the field lines. This effect has been utilized to study electrodeformation of single cells, such as RBCs [10,11], mammalian cells [12], plant protoplasts [13], and cervical cancer cells [14]. Comparing to other experimental methods for cell biomechanics testing, such as classical optical tweezers [15] or micropipette aspirations [16], which are typically complex systems and are limited to measure one cell at a time, DEP can provide much higher throughput analysis through the interdigitated microelectrodes in microfluidic platform [11]. As DEP force is generated by the electrical field in microfluidics, it offers control in both the stress level and loading waveforms [17]. Furthermore, using a theory of membrane viscoelasticity developed by Evans and Hochmuth [18], shear elastic modulus and viscosity of RBC membranes can be determined [19,20,21]. Other models, such as three-stage elastic moduli [15,22] and with an additional dual-viscosity dash pots [19] can be used to describe the nonlinear elastic and viscoelastic behaviors of RBC membranes. The reported values of membrane shear modulus for healthy RBCs were in a range of 2.4–13.3 µN/m [23,24]. 

In a recent article [17], we reported an experimental methodology to study the fatigue behavior of human RBCs in response to a low-cycle DEP fatigue load with a fixed field strength. Cumulative mechanical degradation in cell membranes was observed in both healthy RBCs and Adenosine triphosphate (ATP)-depleted counterparts in response to such cyclic DEP load. To further study the fatigue failure of cell membranes in response to cyclic stress scenarios mimicking the in vivo complexity, it is important to probe the nonlinear elasticity and viscoelastic behavior of RBC membranes using DEP method. Therefore, it is important to investigate the electrodeformation of RBCs that exhibit both small and large deformations. In this paper, we present a method to characterize stress-strain relationship of cell membranes in response to different levels of field strength as well as to extract the nonlinear shear elastic moduli and viscosity. 

## 2. Materials and Methods

**Figure 1:**
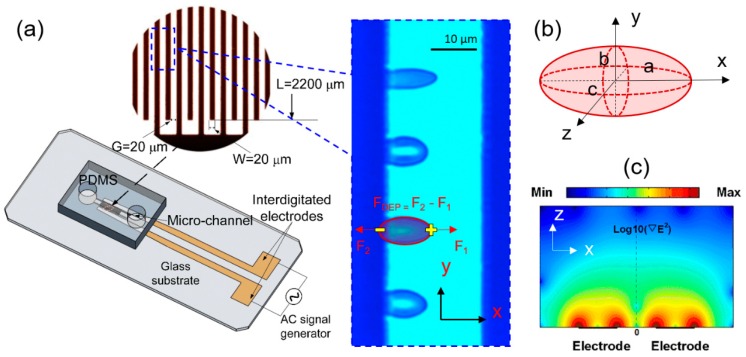
Biomechanics testing of live cells using dielectrophoresis (DEP) in microfluidics. (**a**) Microfluidic device with inset of microscopic view of cellular deformation. Dark strips represent interdigitated electrodes. (**b**) A triaxial ellipsoid model for description of positive DEP-induced uniaxial cell deformation. (**c**) Surface plot of the gradient of field strength square, $\nabla E_{rms}^{2}$ in the microfluidic device, from simulation by COMSOL Multiphysics (COMSOL, Inc., Burlington, MA, USA).

The schematic of the microfluidic device for the biomechanical testing is shown in Figure 1a. It consists of a Polydimethylsiloxane (PDMS) microfluidic channel and a glass substrate printed with thin-film interdigitated gold electrodes (20 µm gap and 20 µm band width). Healthy RBCs were diluted in an isotonic working buffer [25] (8.5% *w*/*v* sucrose and 0.3% *w*/*v* dextrose with the electrical conductivity of 0.018 S/m) for testing. Before the testing, the microfluidic channel was primed with the working buffer containing 5% bovine serum albumin to prevent cell adhesion, then washed with the working buffer to remove excess serum. All experiments were performed at room temperature and under stationary condition. As the direction and magnitude of DEP force are both dependent on the frequency of the electrical signal (Equations (1) and (2)), in current setup, when electrical frequency is below 100 kHz, cells were observed to be pushed away from the electrodes, which failed to provide necessary condition to deform cells; as electrical frequency goes beyond 100 kHz to 1.58 MHz, cells were observed to move towards electrodes and magnitude of DEP force increased; beyond 1.58 MHz, magnitude of DEP force decreases. Therefore, to generate a favorable positive DEP force to deform cell membranes in current setup using lowest voltage levels, sinusoid waveform of 1.58 MHz was selected. Varied voltage levels (0.5–1.0 V_rms_ with an interval of 0.1 V_rms_, 1.5 V_rms_, 2.0 V_rms_, 2.5 V_rms_, 3 V_rms_, and 3.5 V_rms_) were supplied to the electrode array to deform cell membranes. The DEP force field aligned individual RBCs to the field lines and stretched uniaxially (Figure 1a inset and Figure 1b). Each excitation was maintained for 10 s, allowing all cells reach to a steady-state equilibrium state. Cell extension and relaxation process was recorded by a charge-coupled device (CCD) camera attached to an inverted Olympus IX81 microscope (Olympus, Tokyo, Japan) with bright-field imaging. A bandpass filter 414 ± 46 nm was inserted in the light path to improve the image contrast of RBCs. 

RBCs moved toward the higher electric field strength due to the net DEP force from the two electrical force components, *F*_1_ and *F*_2_ exerted on the induced dipole on cell membranes (Figure 1a inset). This net DEP force, *F_DEP_*, has been the primary mechanism widely used for cell trapping and sorting. As cells approach to the edges of electrodes with highest field strength, reaction force from the electrode balances with the DEP force where cells are in transient equilibrium. In this case, RBCs are deformed uniaxially due to the force equilibrium from three components, the reaction force and the electrical force, *F*_2_ in negative *x*-direction, as well as the electrical force, *F*_1_ in the positive *x*-direction. Assuming a stretched RBC as an ellipsoid, the time-averaged DEP force exerted on cell membranes depends on the relative polarizability of the cell and surrounding medium, field strength, cell shape and size and can be determined from [9],

(1) $$\langle F_{DEP}\rangle =2\pi abc\cdot \varepsilon_{m}\cdot Re\left(f_{CM}\right)\cdot \nabla E_{rms}^{2}$$

where *a*, *b*, and *c* are the semi-principal axes of the ellipsoid, $\varepsilon_{m}$ is the permittivity of the surrounding medium, and $\nabla E_{rms}^{2}$ is the root-mean-square value of the gradient of electric field strength square. The value of the real part of the Clausius–Mossotti factor, $f_{CM}$ is determined from the complex permittivity and size of a RBC and its surrounding medium, with a simplified single-shell ellipsoid model [26] according to the concentric multi-shell theory [27],

(2) $$f_{CM}=\frac{1}{3}\frac{\left(\varepsilon_{mem}^{*}-\varepsilon_{m}^{*}\right)\left[\varepsilon_{mem}^{*}+A_{1}(\varepsilon_{cyto}^{*}-\varepsilon_{mem}^{*})\right]+\rho (\varepsilon_{cyto}^{*}-\varepsilon_{mem}^{*})\left[\varepsilon_{mem}^{*}-A_{1}(\varepsilon_{mem}^{*}-\varepsilon_{m}^{*})\right]}{\left(\varepsilon_{m}^{*}+A_{1}(\varepsilon_{mem}^{*}-\varepsilon_{m}^{*})\right)\left[\varepsilon_{mem}^{*}+A_{1}(\varepsilon_{cyto}^{*}-\varepsilon_{mem}^{*})\right]+\rho A_{2}\left(1-A_{2}\right)(\varepsilon_{cyto}^{*}-\varepsilon_{mem}^{*})(\varepsilon_{mem}^{*}-\varepsilon_{m}^{*})}$$

where the subscripts cyto, mem and m stand for cytoplasm, membrane and medium, respectively. $\varepsilon^{*}=\varepsilon -j\sigma /\omega$ with ω as the angular frequency, *ε* and *σ* as the dielectric permittivity and conductivity, respectively. $\rho =\frac{a^{\prime }b^{\prime }c^{\prime }}{abc}$, with $a^{\prime }=a-t,\;b^{\prime }=b-t,\;c^{\prime }=c-t$, *t* as the thickness of cell membrane, 4.5 nm, *c* = 1.3 µm. $\varepsilon_{mem}=4.44$, $\varepsilon_{cyto}=59$, $\sigma_{mem}=10^{-6}\;S/m$, $\sigma_{cyto}=0.31\;S/m$. The depolarization factor along the extensional direction, $A_{i=1,2}$ are defined by

(3) $$A_{1}=\frac{1}{2}abc\int_{0}^{\infty }\frac{ds}{\left(s+a^{2}\right)B}$$

(4) $$A_{2}=\frac{1}{2}a^{\prime }b^{\prime }c^{\prime }\int_{0}^{\infty }\frac{ds}{\left(s+{a^{\prime }}^{2}\right)B^{\prime }}$$

where $B=\sqrt{\left(s+a^{2}\right)\left(s+b^{2}\right)\left(s+c^{2}\right)},\;B^{\prime }=\sqrt{\left(s+{a^{\prime }}^{2}\right)\left(s+{b^{\prime }}^{2}\right)\left(s+{c^{\prime }}^{2}\right)}$ and *s* is an arbitrary distance for integration.

RBC exhibits viscoelastic behavior in response to an external force. The shear stress–strain relationship of cell membranes was considered to be linear for small deformations and nonlinear for large deformations [28]. The membrane shear modulus is relevant to both shear strain and strain rate, especially for large deformations [15,29]. When the external load is removed, the deformed cell recovers to its original shape in a time-dependent manner, due to the elastic energy storage and viscous dissipation in the membrane. A classical model to explain the viscoelastic behavior of the cell membranes was developed as an analogue of the classical Kelvin–Voigt solid model [30],

(5) $$T_{s}=\frac{\mu }{2\left(\lambda^{2}-\lambda^{-2}\right)}+\frac{2\eta }{\lambda }\frac{\partial \lambda }{\partial t}$$

where $T_{s}$ is the shear stress, λ is the stretch ratio, μ and η are membrane shear elastic modulus and viscosity. In this study, the value of $T_{s}$ is calculated by *F/2b*, where *F* is assumed as *F_DEP_*/2 [12,31]. 

## 3. Results

**Figure 2:**
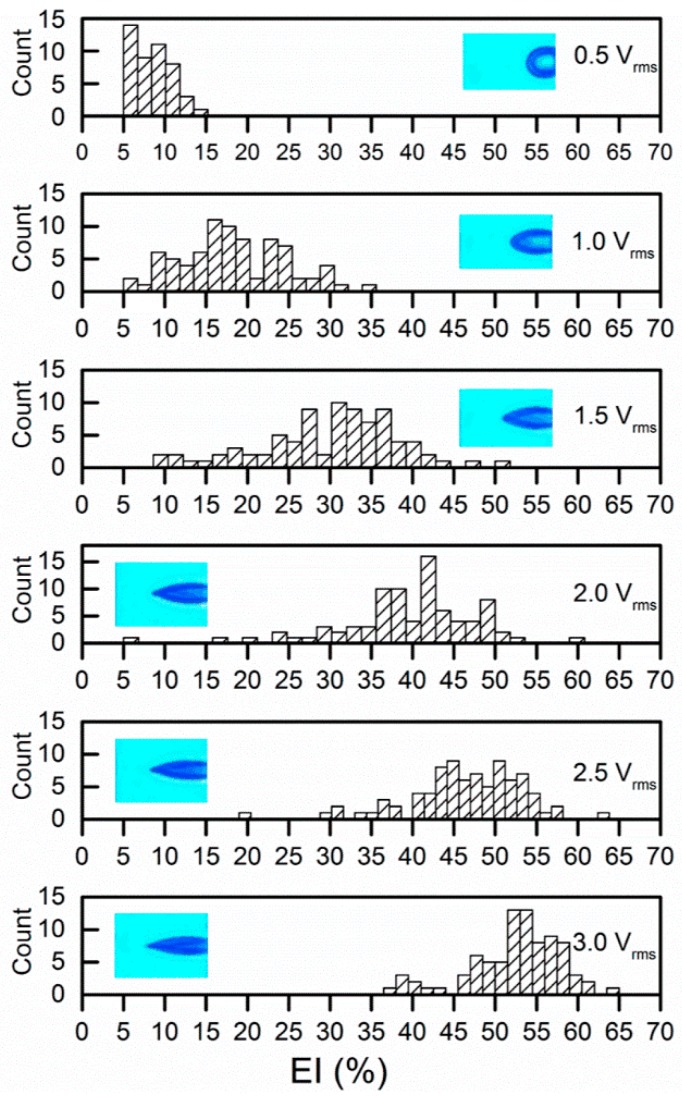
Electrodeformation *EI* values of red blood cells (RBCs) at a series of voltage levels with insets of representative deformed cells.

Cell membrane deformation was extracted by a custom, MATLAB-based (The MathWorks, Natick, MA, USA) imaging processing program. Two parameters were utilized to quantify the membrane deformation, including extensional index, *EI*%= (*a* − *b*)/(*a* + *b*) and extension ratio, *λ* = *b*/*b _t_*_= 0_. The values of *EI* measured at the stationary state for RBCs (*n* = 84) increased with the strength of the applied electric field (Figure 2). The average values of *EI* were 5.6% ± 3.9% to 17.5% ± 7.1%, 29.6% ± 8.9%, 39.5% ± 8.4%, 46.4% ± 7.09%, 52.4% ± 5.6%, respectively. 

**Figure 3:**
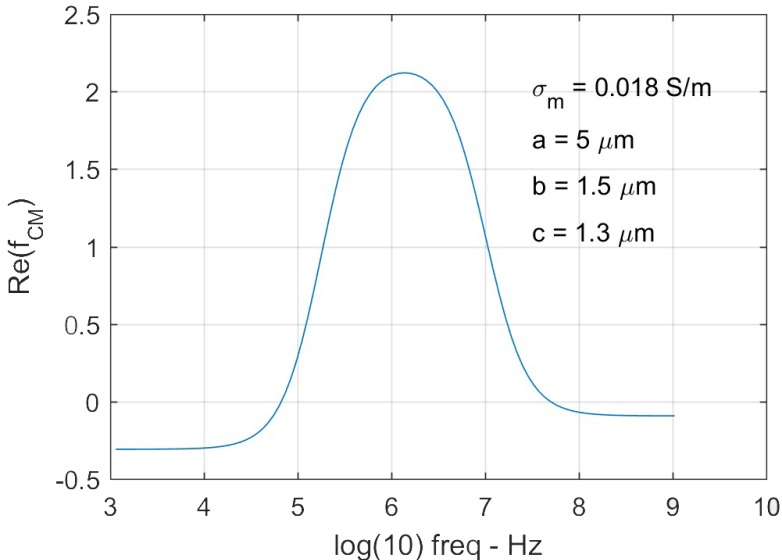
A representative Clausius-Mossotti (CM) factor of a deformed RBC based on the triaxial ellipsoid multi-shell model. The semi-principal axes of the cell are *a* = 5 µm, *b* = 1.5 µm, and *c* = 1.3 µm.

**Figure 4:**
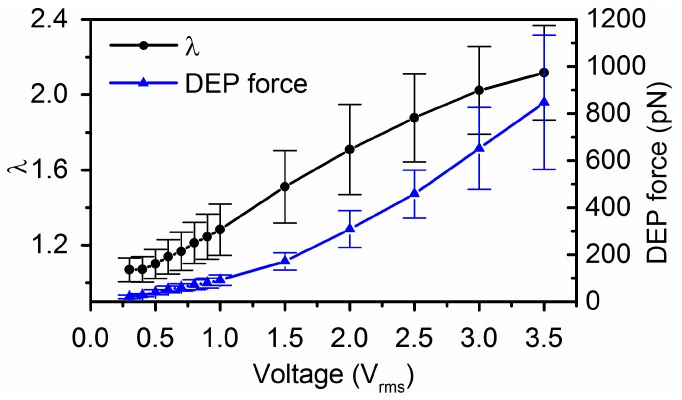
Electrodeformation of RBCs (*n* = 84) with estimated DEP force magnitude and the corresponding extension ratio, *λ* as functions of applied voltage levels. Error bar indicates standard deviations.

The magnitude of DEP force was determined using Equation (1), where *a*, *b* and *c* are the semi-principal axes of the ellipsoid (Figure 1b), $\nabla E_{rms}^{2}$ is the gradient of the root-mean-square (rms) value of the field strength square (Figure 1c). We tracked 84 cells of their equilibrium deformations individually at each field strength in order to determine the DEP forces exerted on the individual cells. As a cell deforms in response to electric excitation, the value of $f_{CM}$ varies with time and the electrical frequency according to Equation (2). A representative value of $Re\left(f_{CM}\right)$ for a deformed RBC is shown in Figure 3. The maximum value of $Re\left(f_{CM}\right)$ was found to be around the selected electrical frequency, 1.58 MHz. Values of extension ratio, λ as a function of time for individual RBCs were characterized experimentally. Mean values of the DEP force magnitude and the corresponding extension ratio, *λ* were plotted as functions of applied voltages (Figure 4). Then membrane shear modulus, *µ* was determined from the relationship between the maximum stretch ratio and the corresponding shear stress using Equations (1) and (5), when $\frac{\partial \lambda }{\partial t}$ approaches to zero. 

**Figure 5:**
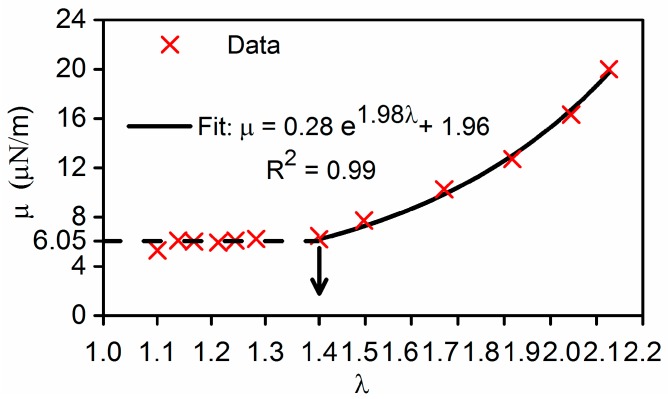
Mean values of membrane shear modulus and best fit functions for nonlinear elastic moduli: for small deformation (*λ* < 1.4), membrane shear modulus, *µ* = 6.05 µN/m; for large deformation (*λ* > 1.4), $\mu =0.28\times e^{1.98\lambda }+1.96$

Nonlinearity in membrane shear moduli was observed, which can be expressed as a combination of two separate functions (Figure 5). For small deformations *λ* < 1.4, membrane shear modulus, *µ* is approximately a constant, 6.05 ± 0.33 µN/m. For large deformation *λ* > 1.4. The value of *µ* can be well fitted with an exponential function with respect to the extension ratio, $\mu =0.28\times e^{1.98\lambda }+1.96$. When *λ* approaches to 2.1, value of *μ* was found to be 20.85 µN/m. These results are in the range of reported literature values, obtained by other independent studies. Membrane shear modulus obtained by other DEP study [32] was in the lower range of 1.4–2.5 μN/m. The nonlinear shear moduli of RBC membranes determined from a sophisticated 3D finite element cell model and optical tweezers experiments [15] were 2.4–5.0 µN/m for small deformations, and 5.3–11.3 µN/m for large deformations, and 13.9–29.6 µN/m prior to failure. The discrepancy between our measurements and other studies may be attributed to several factors, such as the constitutive models used, variations in samples, experimental conditions, optical resolution of the measuring systems, and force calibration among various studies. 

**Figure 6:**
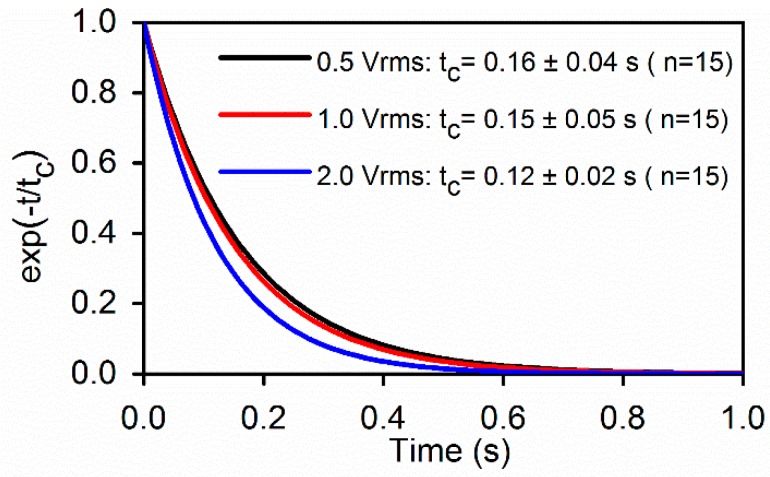
Best fit to the extensional recovery of RBC membranes (*n* = 15). The averaged characteristic time was found to be *t_c_* = 0.14 ± 0.05 s for the three voltage levels.

Upon a sudden release of DEP load, stretched RBCs recovered to their stress-free state at a time-dependent rate. Membrane viscosity, *η* was determined from the extensional recovery when $F_{DEP}$ was deactivated (*T_S_* = 0), based on Equation (5) following the normalization method developed by Hochmuth [30]. Figure 6 compares the *t_c_* values of cell membranes determined from RBCs initially stretched at different levels of field strength. Value of *t_c_* was 0.14 ± 0.05 s, which compares well with the value 0.19 s measured using optical tweezers method [33]. From *t_c_* ≡ *η*/*µ*, shear viscosity of cell membrane can be determined, which is 0.8–2.9 (µN/m)·s, which are consistent with the reported standard values obtained by micropipette aspiration, 0.6–2.7 (µN/m)·s [24] and by optical tweezers, 0.3–2.8 (µN/m)·s [15].

.

## 4. Discussion

Comparing to other DEP studies, discrepancy may mainly arise from assumptions of the shape of deformed cells. Spherical model (*a* = *b* = *c*) has been widely used to estimate the force exerted on bioparticles. Considering many bioparticles are highly nonspherical, such as DNA strands and *E. coli*, ellipsoid models, such as prolate and oblate, have been developed which can provide an improved accuracy for force calibration [34]. In the case of RBC deformation, spherical model is no long valid; the prolate assumption may be valid for large deformations but may overestimate the DEP force for small deformations. Considering the magnitude of DEP force is proportional to the volume of the deformed cell and usually b value is higher than c in uniaxially stretched RBCs, an ellipsoid (a≠b≠c) model can provide an improved accuracy for fore calibration, comparing to the spherical and prolate models. On the other hand, tip formation was noticed in RBCs, which exhibited large deformations at high electric field strengths (Figure 2). For the same reason, even though the triaxial ellipsoid model may be very close to an uniaxially deformed RBC, it can still overestimate the DEP force to some extent. Consequently, the characterized shear modulus for large deformations may be slightly higher than the true values. A numerical solution using finite element model shall be used to account for such variation, as pointed out by an earlier study [20].

## 5. Conclusions

In summary, we performed a systematic electrodeformation study of human RBCs and characterized the nonlinear elastic moduli using the relationship between shear stress and membrane deformation of cell membranes stretched by different electric strengths. The extensional recovery characteristic time of cell membranes was found to be a constant from the extensional recovery processes of RBCs that were initially deformed to different levels. We envision that the general ellipsoid model and the microfluidic DEP platform can benefit biomechanical studies of other cell types and diseased cells.
